# Heterogeneity of diffusion tensor imaging measurements of fractional anisotropy and mean diffusivity in normal human hearts *in vivo*

**DOI:** 10.1186/1532-429X-17-S1-O1

**Published:** 2015-02-03

**Authors:** Laura-Ann McGill, Andrew D Scott, Pedro Ferreira, Sonia Nielles-Vallespin, Tevfik F Ismail, Philip J Kilner, Peter Gatehouse, Sanjay K Prasad, Archontis Giannakidis, David Firmin, Dudley J Pennell

**Affiliations:** 1 The Cardiovascular BRU, Royal Brompton, Sydney Street, UK; 2 National Institutes of Health, National Heart, Lung and Blood Institute, Bethesda, MD, USA; 3 The NIHR Cardiovascular BRU Royal Brompton Hospital, London, UK; 4 NHLI, Imperial College London, London, UK

## Background

Cardiac diffusion tensor imaging (cDTI) by cardiovascular magnetic resonance is becoming more robust for clinical imaging. It has the potential to assess microstructural changes, such as disarray in hypertrophic cardiomyopathy, through measures of fractional anisotropy (FA) and mean diffusivity (MD).[1] However, normal variation in regional and transmural FA and MD is not well described.

## Methods

Twenty normal subjects were imaged on a 3T Siemens Skyra scanner using a breath hold, mono-polar, diffusion weighted STEAM EPI sequence, as previously described.[2] Imaging was performed in a short axis slice of the mid left ventricle, during the systolic pause, with an optimised protocol (b_main_ 750, b_ref_ 150 s/mm^2^).[3]

## Results

**Figure 1:**
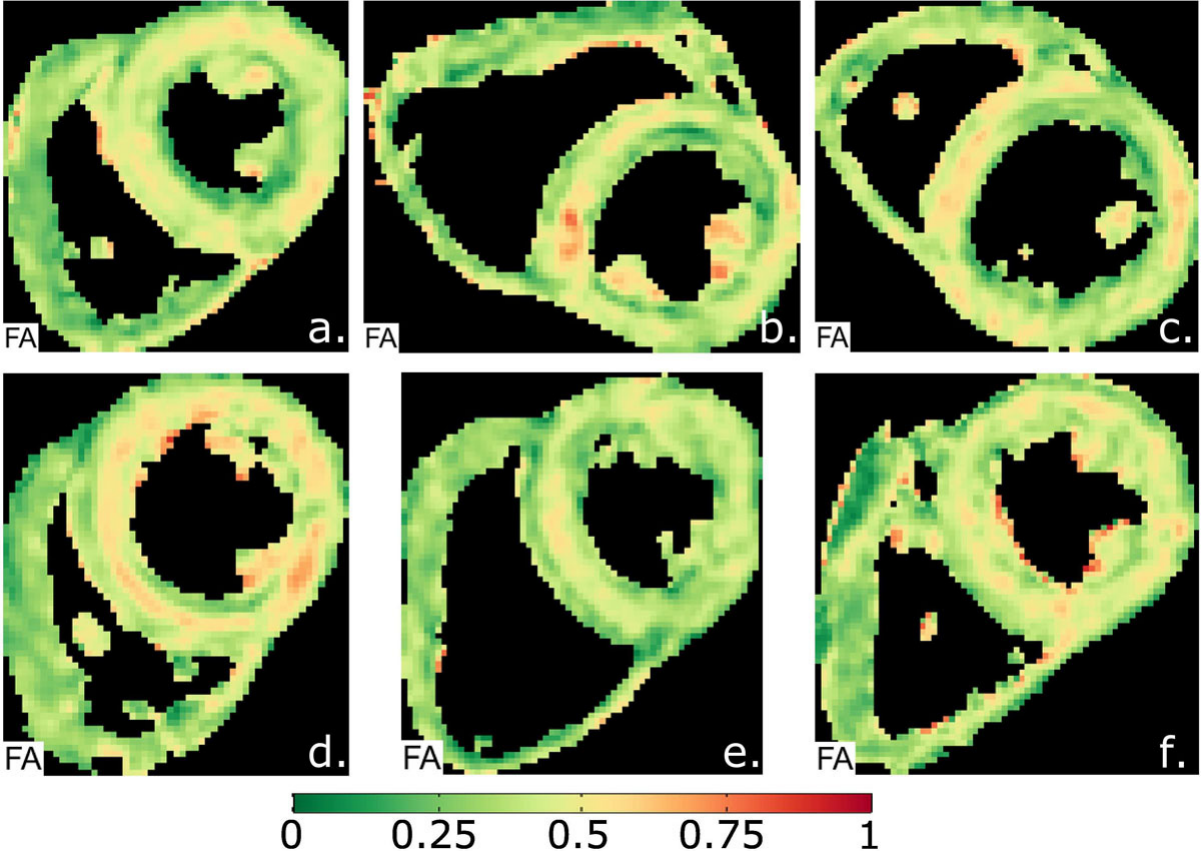
Typical examples of fractional anisotropy (FA) maps, which show a circumferential increase in FA (red) in the mesocardium, indicating a more anisotropic composition of fibres.

**Figure 2:**
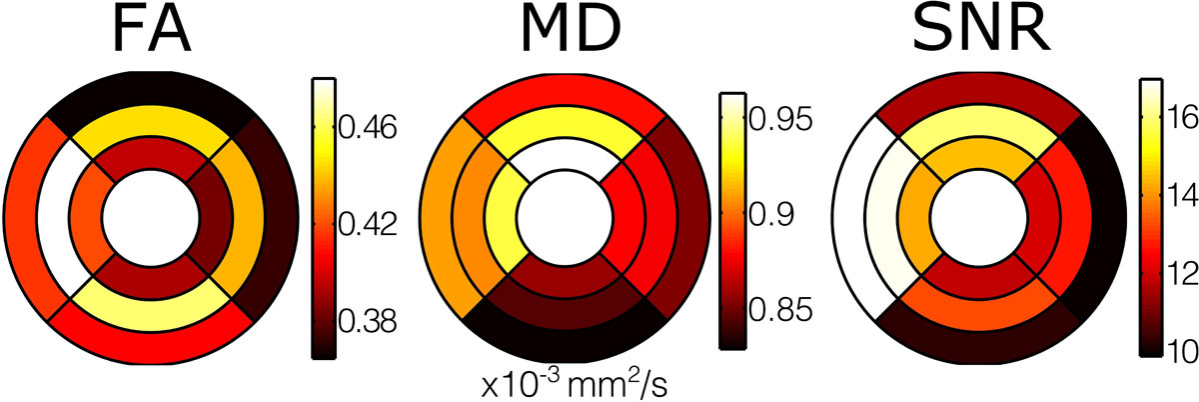
Colour bullseye maps showing significant heterogeneity of average FA, MD and SNR measurements both regionally and transmurally. The outer ring shows results from the epicardium, the middle ring the mesoocardium and the inner ring shows the endocardium, of the single left ventricular slice that was imaged. The upper wall is anterior; right - lateral wall; lower - inferior wall; and left - septal wall.

FA was higher in the mesocardium (0.46 ±0.04) than the endocardium (0.40 ±0.04, p≤0.001) and epicardium (0.39 ±0.04, p≤0.001) (figure 1). There was no difference between endocardial and epicardial FA (p=1.0). On regional analysis, the FA in the septum was greater than the lateral wall (0.398 ±0.05 vs 0.439 ±0.03 p=0.04). There was a transmural gradient in MD (epicardium 0.87 ±0.07; mesocardium 0.89 ±0.07; endocardium 0.91 ±0.08 ×10^-3^ mm^2^/s) with MD greater in the endocardium than epicardium (p=0.04). With the lateral wall (0.87 ± 0.08×10^-3^ mm^2^/s) as the reference, the MD was higher in the anterior wall (0.924 ±0.07×10^-3^ mm^2^/s, p=0.016) and septum (0.915 ±0.07×10^-3^ mm^2^/s, p=0.028). The signal to noise ratio (SNR) was greater in the mesocardium (14.4 ±2.46) than the endocardium (12.9 ±2.14, p<0.001), and epicardium (12.0 ± 2.35, p<0.001). Regional analysis showed the SNR of the lateral wall (11.5 ±1.46) was lower than the septum (16.0 ±3.43, p<0.001) and anterior wall (14.0 ±3.10, p<0.001) (figure 2). The primary eigenvalue in the mesocardium (1.33 ± 0.08×10^-3^ mm^2^/s) was greater than in the endocardium (1.28 ± 0.09×10^-3^ mm^2^/s, p=0.001) and epicardium (1.24 ± 0.07×10^-3^ mm^2^/s^1^, p<0.001). Transmural analysis of helical angle (HA) suggested a plateau in the mesocardium, with a reduced gradient.

## Conclusions

FA and MD measurements are heterogeneous, varying significantly transmurally and regionally in the normal heart. Contributors to this heterogeneity are many, complex and interactive but may include SNR, variations in cardiac microstructure, partial volume effects and strain. These data indicate that the potential clinical use of FA and MD would require measurement standardisation by myocardial region and layer, unless pathological changes substantially exceed the normal variation identified.

## Funding

The NIHR Cardiovascular Biomedical Research Unit at the Royal Brompton Hospital & Imperial College London.
